# Migrant Somali women’s experiences with their first contact with the labor ward prior to admission: A qualitative study

**DOI:** 10.18332/ejm/150584

**Published:** 2022-07-21

**Authors:** Eline S. Vik, Randa M. A. Hashi, Maryam E. Hamud, Vigdis Aasheim, Tone Kringeland, Katrine Aasekjær

**Affiliations:** 1 Faculty of Health and Social Sciences, Western Norway University of Applied Sciences, Bergen, Norway

**Keywords:** birth, experiences, migrant, Somali women, pre-hospital care, first contact

## Abstract

**INTRODUCTION:**

The first contact with the labor ward is a crucial moment in any birth and can be especially challenging when the woman has a migrant background. The aim of the study was to explore migrant Somali women’s experiences with their first contact with the labor ward prior to admission, in Norway.

**METHODS:**

In this qualitative study, data were collected through semi-structured individual interviews and Systematic Text Condensation was the method used to analyze the data. Ten Somali-born women who had given birth in Norway were included.

**RESULTS:**

The migrant Somali women’s first contact with the labor ward prior to admission was challenging, due to one-way communication, poor health literacy, and a fear of interventions or adverse outcomes. To improve care, the migrant Somali women highlighted a need for: 1) flexibility, tailored information and practical guidance before birth; and 2) face-to-face assessment in early labor.

**CONCLUSIONS:**

The findings in this study indicate that the needs of laboring migrant Somali women are not fully met by antenatal or pre-hospital maternity care services. To improve the critical first contact with the labor ward for migrant Somali women, this study suggests that antenatal care services offer practical guidance on whom to contact and what to expect at the hospital in early labor. Face-to-face assessment of maternal and fetal well-being should be the first choice of care for Somali women in early labor who are unfamiliar with the healthcare system after immigration. Interpretation services need strengthening and our findings support a need for increased continuity of care.

## INTRODUCTION

The first contact with the labor ward is a crucial moment in any birth^1,2^. Being a migrant can be considered a risk factor in itself for poor pregnancy outcomes, highlighting how the first contact can be especially crucial when planning care for migrant women^3^. During the first contact, healthcare professionals assess the medical needs of both mother and baby and decide whether the pregnant woman should be admitted, referred to a higher level of care, or await admission^1^. It is not uncommon for migrant women to experience their first encounter with their new country’s healthcare system in relation to pregnancy or childbirth^4^.

According to the United Nations High Commissioner for Refugees, nearly 1 million Somalis have been forcibly displaced by war, persecution, or human rights abuses, often for generations^5^. Compared to the host population, Somali women have been identified with increased risk of a range of adverse outcomes, such as caesarean section^6^ and stillbirth^6,7^. A poor pregnancy outcome in migrant women may depend on a range of factors, including language barriers^3^, lack of trust in the healthcare system^8^, incomplete medical records^3^, giving birth unplanned out-of-hospital^9^, or poor health literacy^10^. There is a need for addressing inequity in maternity care in Europe, especially in relation to migrant women^3,7^.

Studies have shown that laboring women often negotiate their credibility before being admitted to hospital^11^ and women tend to be encouraged by midwives to stay home for as long as possible prior to admission^11^. The World Health Organization expresses skepticism towards a policy of delaying labor ward admission for women presenting in spontaneous labor^2^ and guidelines in intrapartum care pay little attention to the diverse needs of migrant women^12^. To our knowledge, no similar studies have been conducted related to migrant Somali women’ experiences with early labor and admission to labor ward in Norway or similar settings. Therefore, we set up a study to explore Somali women’s experiences with giving birth in Norway and their experiences with the contact they had with the labor ward prior to admission.

## METHODS

This is a qualitative study. The RATS Checklist (Relevance, Appropriateness, Transparency, and Soundness) was used for strengthening the reporting of the data^13^.

### Setting

In Norway, maternity care is available for all women independent of legal status, ethnicity or social background^14^. However, regardless of migrant women’s rights, some migrant women who have given birth in Norway have described being treated with indifference or a lack of respect^15^, and some migrant Somali women have expressed distrust of Norwegian antenatal care^8^. Somalis represent one of the largest migrant groups in Norway, and Somali mothers give birth to more children than the national average^16^. Midwives are the main caregivers in labor wards.

### Participants

Women eligible for the study were Somali-born women aged ≥18 years who had given birth in Norway within the last ten years. Data were collected through October 2018, and a total of ten Somali women were included. Characteristics of the participants are shown in Table 1. Altogether, the women shared their experiences from 28 births in Norway ranging from one to six births per woman. Three women had given birth to their firstborn child in Somalia, a topic not included in this study.

**Table 1:** Characteristics of the participants, Norway, 2018 (N=10)

*Characteristics*	*n*
**Age** (years), mean (range)^a^	31 (26–38)
**Parity**	
Nulliparous	2
Parous, (number of births)^a^	8 (2–6)
**Place of first birth**	
Somalia	3^b^
Norway	5
**Years of stay in Norway**, mean (range)^a^	12 (5–25)
**Understanding of the Norwegian language^c^**	
Fluent	2
Understood some Norwegian	4
Understood little or nothing	4^d^

a At time-point of interview, not time-point of birth.

b One firstborn child born in Somalia died in Somalia before migration to Norway.

c At time of the last birth in Norway, based on self-reported data.

d Two reported that they understood some English.

### Data collection

Data were collected through semi-structured individual interviews. At the time of the interviews, the women lived in eastern and western parts of Norway, and they were interviewed in their home or in a place chosen by the women. Seven interviews were conducted face-to-face, and three over the telephone due to long distances. Initially, women eligible for the study were recruited through social media and by contacting Somali associations in Norway. Migrant Somali women may, however, represent a hard-to-reach population in health research^17^. Therefore, after the initial inclusions, we changed to a snowball sampling strategy^18^. In line with the study ethics, the participants were not given incentives to take part in the study.

The authors RMAH and MEH are bilingual (i.e. fluent in both Norwegian and Somali) with enhanced knowledge of the Somali community in Norway. These two authors conducted the interviews enabling the women to speak freely in their preferred language. Based on the women’s choice, two interviews were conducted in Norwegian and eight interviews in Somali. To include all co-authors in the process of analyzing the data, RMAH and MEH transcribed and translated all interviews into Norwegian.

The interview guide consisted of six open-ended questions and five background questions (Table 2). The interviews started with the following question: ‘Could you please tell us about your first birth experience in Norway?’ and ‘Please, start at the moment when you realized that the birth had started, before you contacted your planned place of birth’. The women were asked to focus on the moment they realized that the birth had started and include in their description the time they stayed at home before contacting the delivery ward. Each interview lasted from 15–40 minutes (mean: 26), and the open-ended questions focused on Somali women’s experiences with giving birth in Norway and their experiences with the contact they had with the labor ward prior to admission.

**Table 2:** Interview guide, Norway, 2018

*No*	*Questions*
1	Could you please tell us about your first birth experience in Norway? Please, start at the moment when you realized that the birth had started, before you contacted your planned place of birth.
2	Could you tell us about the first contact you had with your planned place of birth? How did you contact your planned place of birth and how did you experience the contact?
3	Were you in contact with your planned place of birth more than once while pregnant or in labor? If so, can you tell us about it?
4	Could you tell us about what you were thinking and what you did when it became clear that it was time to go to the hospital?
5	Could you tell us about your first meeting with the midwife who welcomed you when you arrived at the hospital?
6	Is there anything else you would like to add?

### Analysis

Systematic Text Condensation (STC)^19^ was used to analyze the data. STC consists of four steps. Firstly, we read the interviews to establish an overview of the data looking for preliminary themes. In this step, eight themes relevant to the aim of the study were identified. In the second step, we identified meaning units in the text and reduced the initial eight themes to five preliminary code groups reflecting our increased understanding of the material. In the third step, the meaning units of each code group were sorted into a limited number of subgroups. At this stage, the five temporary code groups from step two were reduced to the final three code groups on which our final analytical text is based. Further, the meaning units of each subgroup were condensed and summarized into a condensate written in first person voice. This was a flexible process where we went back and forth in the original data material aiming to identify and organize data elements in line with the aim of the study. In the fourth step of the analysis, we re-contextualized the text representing each subgroup resulting in a final analytic text written in a third person perspective. Finally, we identified authentic quotations from the interviews that could illustrate the content of each subgroup.

## RESULTS

The migrant Somali women’s first contact with the labor ward prior to admission was challenging, due to one-way communication, poor health literacy, and a fear of interventions or adverse outcomes. To improve care, the migrant Somali women highlighted a need for: 1) flexibility, tailored information and practical guidance before birth; and 2) face-to-face assessment in early labor.

### One-way communication

All women in the current study were in contact with the hospital prior to admission, either by phone or appointment. The calls were described as being in an interview situation, rather than a dialog between two equal individuals. The women were grateful for the way they were met on the phone and described the healthcare professionals as open and helpful in their approach. Even so, the women said they were asked difficult questions that they did not necessarily understand. Those who spoke Norwegian well said they got adequate information from the healthcare professionals. However, most women in this study had limited language skills, and they expressed how language barriers complicated their contact with the labor ward. None of the women in this study was offered a professional interpreter in the encounter with the labor ward, but some had a friend or a family member with them who helped with communication. One first time mother explained how language barriers affected her encounter with the labor ward:

*‘The greatest challenge I have experienced is the following: If you are incapable of understanding anything [they say], then no one can help you. I did not have good enough language skills to express myself. I was not offered an interpreter, and I never got the help I needed.’* (Interview 10)

### Poor health literacy, a fear of interventions and adverse outcomes

The women described labor pain as a natural part of childbirth, something that women must handle. All participants spoke of their knowledge about caesarean section and pain relief in labor. Especially caesarean section was spoken of as something associated with high levels of risk. One woman said she was afraid the doctors would steal a kidney from her if they had performed a caesarean section on her, and another woman said she had heard that there could be a 50% chance of not surviving the operation. The misinformation on the negative effects of caesarean section came from other Somali mothers. Other concerns learnt from Somali mothers included a risk of becoming disabled after interventions or that an epidural could possibly prolong labor or hinder labor progress. This kind of information made the women worried, and some said they therefore refused interventions or help with labor pain during labor. One parous woman explained it in the following way:

*‘I was afraid they would put a needle in my back. I had heard from other Somali women that the needle was bad for me, and that the procedure can leave you paralyzed [for life].’* (Interview 7)

The women talked about their knowledge in relation to childbirth and the onset of labor. The knowledge they expressed varied in content. However, most of the women expressed a genuine fear of adverse pregnancy outcomes and that the birth would not develop as expected. The women explained how their perceptions of childbirth were mainly based on knowledge shared by family members or friends. Some said their general practitioner or midwife had given them information about the onset of labor, while others said most information came from their own network. Their knowledge was also based on information from books, or from their own experiences related to pregnancy and childbirth. In cases where the onset of labor started in a different way than they expected, the women expressed concerns about the well-being of the baby, like this first-time mother:

*‘I felt concerned when I saw the amniotic fluids, because this was my first baby … I had read that if the water broke, the baby could get the umbilical cord around its neck and die.’* (Interview 8)

### A need for flexibility, tailored information and practical guidance before birth

The women stressed the importance of finding practical solutions and wished that the healthcare system would be more flexible and health professionals open minded in the encounter with Somali women. Most of the women we interviewed did not have access to a private car, and therefore came to the hospital by taxi or bus. Further, parous women had to arrange for someone to look after older children before they could go to the hospital. The women suggested that strengthening guidance before giving birth would have saved both laboring women and healthcare professionals of unnecessary stress. The amount of information provided was extensive, but the women said it was hard for them to understand the content. When information was only partly available or understood, this led to feelings of distress or misunderstandings. One first-time mother put it like this:

*‘One should get more information before giving birth. I would have liked someone to tell me where to go, what happens at the hospital and all such things … what to expect at the labor ward.’* (Interview 2)

### A need for bridging the gap between themselves and the new healthcare system

Most of the women included in this study considered themselves as newly arrived migrants at the time of labor. The women explained a stressing factor of being unfamiliar with the healthcare system and several were surprised by different elements of practice. After arriving in Norway, the women explained that skepticism towards and unfavorable stories about the Norwegian healthcare system were common among the Somalis they met in Norway. These negative stories from others in the Somali community made them skeptical of the system and healthcare providers. All the women spoke of their family back home in Somalia, and said they missed their network after migration. They missed someone close to share the experiences of childbirth with, and the women who had been the shortest amount of time in Norway said they had given birth without having someone they knew with them. The women spoke of challenges related to cultural insensitive encounters, such as when they were prohibited from bringing their extended family to the labor ward. The women explained how both family and friends were expected to support new families and laboring women, while this practice was less common in Norway. The women described feelings of loneliness, fear and said they did not feel safe in their new situation. Newly arrived pregnant women explained that they were offered extra follow-ups, both at their doctor’s office and at the hospital. However, some women described that language barriers generated fear and hampered optimal care. One woman who gave birth to her first child in Somalia, and was now pregnant with her second baby in Norway, explained how difficulties with communication lead her to be sent to the hospital in an ambulance:

*‘I met my doctor for the first time when I became pregnant. … He asked me if I had been treated for syphilis. I said no. I had never heard of this illness. After the results of blood tests came back, I was sent to the hospital in an ambulance because they were worried about the baby's health. … I was scared to death. When I arrived at the hospital, they had found out that the doctor at the asylum reception center had in fact treated me for syphilis. I did not lie to my doctor, but I thought it was a different illness they had treated me for.’* (Interview 5)

Some of the women brought female friends with them to the labor ward, but the healthcare workers often sent these friends away. The women who experienced that their needs were met regarding whom they wanted to accompany them when admitted said they appreciated the support given by the caregivers. One woman, who had a female friend with her during labor, explained how this empowered her and strengthened her relationship with the caregivers:

*‘I swear, they treated me really good. I have been praying for them. They were good people … I did not understand what they said, but I had a friend with me, and she translated it for me.’* (Interview 5)

### A need for face-to-face assessment in early labor

The women were afraid of being rejected on the telephone and were afraid they would be asked to stay at home. Being rejected was related to the fear of adverse pregnancy outcomes such as unplanned birth at home, bleeding to death, or worry that the baby could die if they were told to stay at home. They said they trusted their own instincts and felt a strong need to protect themselves and their unborn baby. Parous women explained how they recognized signals from their body telling them that labor was in progress. First time mothers said they felt welcomed to come to the hospital, and they said they thought this was due to them expecting their first baby. The women spoke of birth as a natural, but possibly fatal event, and they stressed that all women should be offered a face-to-face assessment of maternal and fetal well-being when awaiting admission to the labor ward. The women were prepared to be sent home again if both mother and fetus were found to be in good health. One parous woman explained her strategy in making sure she would get a face-to-face assessment when her contractions started:

*‘My contractions were not very strong, but I said they were stronger than they actually were … because I was afraid, they would say I had to stay at home. If I had told them the contractions were more than 5 minutes apart, they would have told me to stay at home and then I would have given birth at home.’* (Interview 7)

## DISCUSSION

In the current study, the migrant Somali women’s first contact with the labor ward prior to admission was challenging, due to one-way communication, poor health literacy, and a fear of interventions or adverse outcomes. To improve care, the migrant Somali women highlighted a need for flexibility, tailored information and practical guidance before birth, and face-to-face assessment in early labor.

### Pre-admission challenges when caring for migrant Somali women in early labor

Earlier studies from Norway and the UK have found migrant women to be grateful for the care they receive in the host country^20,21^. This study adds that one-way communication, poor health literacy, and a fear of interventions or adverse outcomes affected the women’s encounters with the labor ward negatively, suggesting that the needs of laboring migrant Somali women are not fully met by current pre-hospital maternity care services. A number of studies yield similar results related to migrant women and language barriers^16,20,21^, varied levels of health literacy^10,22,23^, and fear of interventions and complications^16,24,25^. Similar to our results, a recent cross-sectional study from Norway found that one-third of recent migrant women did not understand the information provided by the healthcare providers during maternity care^21^. The migrant Somali women in the current study described the first contact with the labor ward as an interview situation. This was also found in a British study describing how midwives tended to be more focused on information gathering rather than listening to identify information the women wished to impart^26^. In line with the findings of a recent systematic review focusing on migrant women’s experiences of pregnancy, birth and maternity care in Europe^27^, pre-hospital maternity care can be improved by implementing new models of care based on increased continuity of care for migrant Somali women.

Traditionally, pregnant women in early labor have been encouraged to stay at home as long as possible^11^. A common argument for encouraging women to stay at home is that hospital admission during early labor may increase the risk of unnecessary medical interventions and complications^28^. However, when discussing the needs of migrant Somali women in particular, a risk of unnecessary interventions may be less relevant. Firstly, the women themselves express skepticism against interventions; in the current study, women were afraid that having an epidural would prolong labor. One quantitative Norwegian study showed that Somali women in Norway use less epidural for labor pain compared to Norwegian-born women^29^. As discussed in the Norwegian study^29^, midwives and doctors should pay attention to migrant women’s needs for assistance with communication during childbirth to ensure migrant women are adequately informed about their options. In the current study, labor pain was described as a normal and natural part of birth. This finding is supported by a cross-national qualitative study describing how Sub-Saharan women are determined to stay strong during labor and accept labor pain as natural, based on their maternity experiences from their home country^22^. Similarly, an Australian study reports that migrant women prefer a natural delivery free of unnecessary medical interventions^30^. Secondly, when compared to the host population, Somali women in Norway have been identified with an increased risk of adverse pregnancy outcomes, such as stillbirth^7^. Perinatal mortality rate has been shown to be lower for migrant Somali women in Norway compared to non-migrant Somali women giving birth in Somalia, suggesting that Somali women in Norway are given quality care compared to the care available in their home country^31^. Negative experiences with the healthcare system in their home country may explain why some migrant Somali women may lack trust and be skeptical towards a more functional healthcare system after immigration^3^. A meta-ethnography including 48 studies on patient safety concludes that effective face-to-face communication plays a key role in patient safety^32^, suggesting that migrant Somali women may benefit from a face-to-face check-up when contacting the labor ward. Our findings therefore suggest a need for implementing face-to-face assessment for pregnant migrant Somali women contacting the labor ward in early labor.

Our findings support the conclusion in a recent qualitative study exploring Norwegian first-time mothers’ information needs in early labor^33^, which however should be adjusted to Somali women’s needs; there is an urgent need for increased knowledge on pregnant Somali women’s information needs pre-admission, including studies exploring how the information should be provided to help pregnant Somali women feel safe during pregnancy, the first contact with the labor ward and birth.

### Other suggestions for improving care for migrant Somali women in early labor

One study from Sweden supports Somali women’s need for tailored information, language-support and flexible routines in maternity care^23^. Flexible routines may include women in labor being encouraged to bring their companion of choice for support, such as their partner^23^, a friend, relative or a multicultural doula. Some newly arrived migrant women may benefit from a multicultural doula who can help with communication and bridge the gap between the birthing woman and the maternity care services in the new country^34^. Pregnant migrant women’s health may further benefit from health professionals training on communication skills, or healthcare systems implementing smartphone apps tailored to migrant women on warning signs in pregnancy^35^.

In line with our findings, a systematic review exploring migrant women’s experiences of pregnancy, childbirth and maternity care in Europe, found that some migrant women in Europe struggle navigating the healthcare system^27^, suggesting practical guidance in pregnancy may improve the assessment for migrant Somali women in early labor. Practical guidance in pregnancy may include someone guiding women who are unfamiliar with the healthcare system on where to go and what to expect at the hospital in early labor. While the amount of information provided to the pregnant Somali women may have been extensive in our study, the information becomes worthless when not understood. There is no consensus for best practice related to the follow-up of pregnant and delivering migrant women, therefore further interventions studies are needed.

### Strengths and limitations

The main strength of this study was that two of the authors are of Somali origin, and we were therefore able to enhance trust and include women with little knowledge of Norwegian or English language. Due to the low number of participants the results must be interpreted with caution. Ten women is a limited number, however, they shared experiences from altogether 28 births after migrating to Norway. A study discussing sample size in qualitative research highlights that the more study-relevant information provided by the sample, the lower the number of participants needed^36^.

## CONCLUSIONS

The findings from this study indicate that the needs of laboring migrant Somali women are not fully met by antenatal or pre-hospital maternity care services. Interpretation services need strengthening, and women in labor should be encouraged to bring their companion of choice for support. To improve the critical first contact with the labor ward for migrant Somali women, this study suggests that antenatal care services offer practical guidance on whom to contact and what to expect at the hospital in early labor. Face-to-face assessment of maternal and fetal well-being should be the first choice of care for Somali women in early labor who are unfamiliar with the healthcare system after immigration. Our findings support a need for increased continuity of care and flexibility when caring for laboring migrant Somali women.

## Data Availability

The data supporting this research are available from the authors on reasonable request.
